# GATA2 Deficiency With Early‐Onset and Progressive Interstitial Lung Disease

**DOI:** 10.1002/rcr2.70165

**Published:** 2025-04-22

**Authors:** Yuriko Sugiura, Takahiro Ando, Hirokazu Urushiyama, Akihisa Mitani, Goh Tanaka, Kenichi Kashimada, Tomohiro Morio, Hidenori Kage

**Affiliations:** ^1^ Department of Respiratory Medicine The University of Tokyo Tokyo Japan; ^2^ Department of Pediatrics and Developmental Biology Institute of Science Tokyo Tokyo Japan

**Keywords:** GATA2 deficiency, inborn errors of immunity, interstitial lung disease, nonsense variant, pulmonary hypertension

## Abstract

GATA2 deficiency is a rare disease caused by germline heterozygous variants. This mutation is known to cause a decrease in haematopoietic stem cells and a decrease in monocytes, dendritic cells, NK cells, and B cells, leading to various diseases such as haematological, infectious, respiratory, and neurological diseases. The most common respiratory diseases are pulmonary alveolar proteinosis, recurrent respiratory tract infections, and pulmonary hypertension. A patient had recurrent infections since her childhood, and in her 20s developed sensorineural hearing loss, interstitial lung disease, and was diagnosed with mental retardation. Bronchoscopy did not reveal the cause of interstitial lung disease. Exome analysis revealed a *GATA2* c.1084C>T p.R362* heterozygous variant. The patient developed pulmonary hypertension as the interstitial lung disease progressed when she was 41 years old and currently requires home oxygen therapy. Early‐onset interstitial lung disease may be a rare phenotype of GATA2 deficiency.

## Graphical abstract

GATA2 deficiency is an autosomal dominant disorder with various manifestations including hematologic disease and inborn errors of immunity. The most common respiratory diseases are recurrent respiratory tract infections, pulmonary alveolar proteinosis, and pulmonary hypertension. We report a patient with early‐onset and progressive interstitial lung disease.

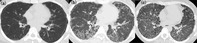

## 1 Introduction

GATA2 deficiency is an autosomal dominant disorder caused by heterozygous variants in the *GATA*2 gene [1]. Pathogenic *GATA*2 variants cause haploinsufficiency [2] and have been reported to cause a variety of diseases, including hematologic neoplasms, inborn errors of immunity (IEI), autoimmune diseases, lung diseases, and neurological diseases [1]. The most common pulmonary diseases are pulmonary alveolar proteinosis (PAP), recurrent respiratory tract infections including nontuberculous mycobacteria, and pulmonary hypertension (PH) [3]. Only one case of interstitial lung disease complicating GATA2 deficiency has been reported [4]. Here, we describe a case of GATA2 deficiency with early‐onset and progressive interstitial lung disease.

## 2 Case Report

A 26‐year‐old woman was referred to our hospital with early‐onset interstitial lung disease. She had a history of recurrent otitis media and infections since childhood, candida esophagitis at age 16, and aseptic meningitis at age 17. At age 23, she was diagnosed with interstitial lung disease, schizophrenia, and mild mental retardation. At age 24, she developed sensorineural hearing loss. Bronchoalveolar lavage performed at the previous hospital was unremarkable, and transbronchial lung biopsy showed non‐specific thickening and fibrosis of the alveolar septum. Her mother had a history of recurrent infections, tuberculosis, Wegener's granulomatosis, and died of hematologic disease at age 45. Her father has a history of mental illness (schizophrenia, bipolar affective disorder, autism) and died of cardiac disease at age 65. She had no siblings.

**FIGURE 1:**
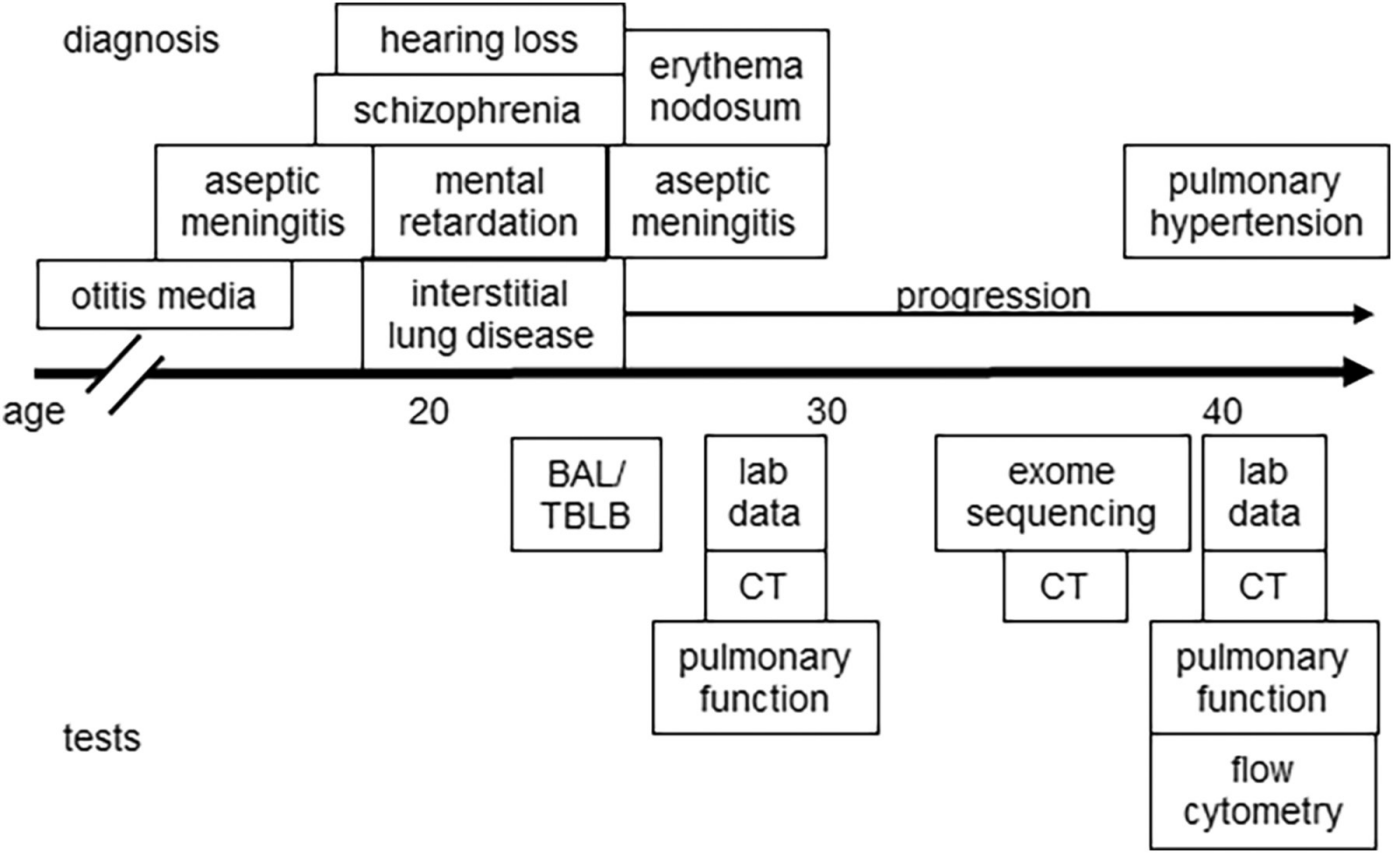
Timeline of events. The events above the arrow are complications and the events below the arrow are the tests that were performed. BAL, bronchoalveolar lavage; TBLB, transbronchial lung biopsy.

**FIGURE 2:**
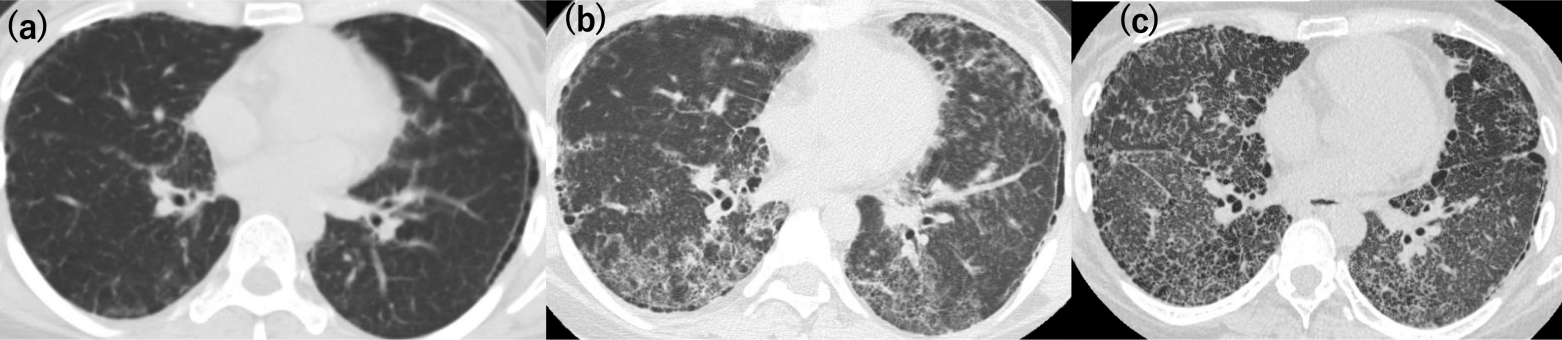
CT images of the lung. (a) age 26: Subpleural reticulation and interlobular septal thickening are noted. (b) age 36, (c) age 41. Disease progression and development of ground‐glass and nodular opacities are noted.

At age 26, she was referred to our hospital for further evaluation. Figure 1 shows the course of events. The patient was serologically negative for antinuclear antibodies (ANA), anti SS‐A antibody, anti SS‐B antibody, MPO‐ANCA, and PR3‐ANCA, and the anti‐measles IgG titre was within normal limits. She was not on any medication and had no environmental or occupational exposures other than cigarette smoking that put her at risk for ILD. An examination by a dermatologist revealed no abnormal findings. Chest CT showed subpleural reticulation and interlobular septal thickening (Figure 2a). At age 28, she developed erythema nodosum and was hospitalised for aseptic meningitis. Her laboratory data revealed a white blood cell count of 2.7 × 10^3^/μL (neutrophil count 1.8 × 10^3^/μL, monocyte count 0/μL, lymphocyte count 300/μL), T cell count 243/μL (82%) and B cell count 11/μL (4%), indicating lymphopenia and decreased B cell count. IgG was normal at 1124 mg/dL. At age 36, exome analysis revealed a nonsense variant in the *GATA2* gene, Chr3(GRCh37):g128200721G>A, NM_001145661.2:c.1084C>T, p.R362*, leading to a diagnosis of GATA2 deficiency. At this time, her white blood cell count was 5.1 × 10^3^/μL (neutrophil count 4.3 × 10^3^/μL, monocyte count 0/μL, lymphocyte count 600/μL) and IgG was 3905 mg/dL. Interstitial lung disease progressed gradually (Figure 2b).

At age 41, she was admitted to our hospital for respiratory failure and exacerbation of heart failure, and cardiac ultrasound showed elevated right ventricular systolic pressure (48 mmHg) suggesting pulmonary hypertension. During the 15 years between referral and admission, interlobular septal thickening with ground‐glass and nodular opacities worsened (Figure 2c), KL‐6 increased from 836 U/mL to 3341 U/mL, forced vital capacity decreased from 2.47 L (94% of predicted value) to 1.29 L (58%), and diffusing capacity decreased from 16.4 (86%) to 5.1 mL/min/mmHg (30%). IgG was 5350 mg/dL. Complete blood count showed white blood cells 4.5 × 10^3^/μL (neutrophils 3.3 × 10^3^/μL, lymphocytes 9.8 × 10^2^/μL, monocytes 41/μL, eosinophils 162/μL, basophils 18/μL). Flow cytometry results showed a decrease in B cells, effector memory T cells, follicular helper T cells, natural killer T cells, and natural killer cells, while dendric cells were deficient (Table 1). The patient was not considered for haematopoietic stem cell transplantation or lung transplantation due to her history of schizophrenia. Prophylactic antibiotics were not required as infections were self‐limiting.

**TABLE 1:** The percentage of different immune cells at age 41.

Leukocyte	4500/μL	
Lymphocytes	980/μL	
CD19+CD20+	0.76%	**
CD20+CD21+	0.46%	
CD20+CD21−	0.32%	*
CD19+CD27+	0.80%	
CD19+CD27−	0.26%	
CD3+	912/μL (93.1%)	
CD4+	457/μL (50.1%)	
CD45RO+	182/μL (39.9%)	
CXCR5+ (follicular helper T)	6.89%	**
CCR6+CXCR3+ (Th1star)	23.8%	*
CD45RO−	68/μL (15.0%)	
CD45RA+CD31+ (recent thymic emigrant)	44/μL (64.5%)	
CD45RA+CD31− (others)	24/μL (35.5%)	*
CD8+	410/μL (45.0%)	
CD45RO+	96/μL (23.4%)	
CD62L+CCR7+ (central memory)	70/μL (73.0%)	
CD62L−CCR7− (effector memory)	11/μL (11.5%)	**
CD45RO−	105/μL (25.7%)	
Va24 + Vb11+ (NKT cell)	0.002%	**
CD16 + CD56+	1.20%	**
Lineage‐HLADR+ CD123+ (plasmacytoid DC)	Almost 0%	**
Lineage‐HLADR+ CD11c+ (classic DC)	Almost 0%	**

*Note:* * abnormally high value, ** abnormally low.

## 3 Discussion

Many variants have been reported in the *GATA2* gene, most commonly in one of the two zinc finger‐binding domains [4]. In this patient, the GATA2 c.1084C>T p.R362* variant was found within the zinc finger II domain. This is a rare variant, as its population distribution frequency has not been reported in public databases such as ClinVar, dbSNP, HGVD, and gnomAD. The ClinVar classification is Pathogenic, and the ACMG pathogenicity rating is considered moderately pathogenic due to the nonsense variant occurring in the second zinc finger‐binding domain.

*GATA*2 variants are known to cause haploinsufficiency [2], resulting in various phenotypic diseases. GATA2 is a zinc finger transcription factor required for the differentiation of endothelial and immature haematopoietic cells. Patients with GATA2 deficiency are at high risk for hematologic malignancies and IEI, and patients with MDS exhibit severe peripheral blood monocytopenia and B cell and NK cell lymphopenia. The *GATA2* c.1084C>T variant has specifically been reported to be associated with MDS, acute myeloid leukaemia (AML), and IEI [2].

In the present case, the patient developed recurrent infection, sensorineural hearing loss, and erythema nodosum, while no hematologic malignancy developed. Based on family history, it is likely that her mother had GATA2 deficiency, and she may have been genetically predisposed to schizophrenia from her father rather than her mother. The patient's history of repeated infections since childhood led us to suspect IEI, which led to the diagnosis of GATA2 deficiency.

Interstitial lung disease is a rare manifestation of GATA2 deficiency [3, 4]. The literature suggests that in general, recurrent infection is a common cause of interstitial lung diseases. In addition, alveolar macrophage dysfunction in GATA2 deficiency may contribute to the development of PAP [5]. However, this patient did not manifest recurrent pneumonia, and CT and bronchoscopy did not suggest PAP. She was not on any medication at her first visit to our clinic, and based on the clinical course and laboratory results, it is unlikely that she had drug‐induced interstitial lung disease or collagen‐related interstitial lung disease. We concluded that this patient's interstitial lung disease was likely caused by GATA2 deficiency.

## Author Contributions

Y.S. collected clinical data and wrote the manuscript. T.A., H.U., A.M., and G.T. contributed clinical data, reviewed, and edited the manuscript. K.K. and T.M. provided genetic information and conducted FACS analysis. H.K. designed the study concept, contributed clinical data, and edited the manuscript. All authors approved the final version of the manuscript for publication.

## Ethics Statement

The authors declare that appropriate written informed consent was obtained for the publication of this manuscript and accompanying images.

## Conflicts of Interest

The authors declare no conflicts of interest.

## Data Availability

The data that support the findings of this study are available from the corresponding author upon reasonable request.
